# The Association Between the Social Determinants of Health and HIV Control in Miami-Dade County ZIP Codes, 2017

**DOI:** 10.1007/s40615-020-00838-z

**Published:** 2020-08-17

**Authors:** Dayana Rojas, Anamarie Melo, Imelda K. Moise, Jorge Saavedra, José Szapocznik

**Affiliations:** 1 Department of Public Health Sciences, University of Miami, 1120 NW 14th ST, Miami, FL 33136 USA; 2 Urban Health Solutions, Miami, FL USA; 3 Department of Geography and Regional Studies, University of Miami, Miami, FL USA; 4 AHF Global Public Health Institute, University of Miami, Miami, FL 33136 USA; 5 Department of Public Health Sciences, University of Miami, Miami, FL USA

**Keywords:** HIV, Social determinants of health, Detectable viral load, GIS

## Abstract

**Background:**

There were 28,055 people living with HIV (PLWH) in Miami-Dade County (MDC) in 2017; 40.1% was either out of care or was not virally suppressed (uncontrolled HIV). The purpose of this study was to determine the association between the social determinants of health (SDOH) and the number of persons with uncontrolled HIV in MDC.

**Setting:**

This cross-sectional study included PLWH 15 and older with uncontrolled HIV in MDC, 2017. Data on PLWH’s viral load, age, gender, mode of HIV transmission, and race/ethnicity were aggregated to the ZIP code level. All five SDOH per HealthyPeople 2020 were represented: economic stability, education, social and community context, health and healthcare, and neighborhood and built environment.

**Methods:**

Descriptive analyses on all study variables and a principal component analysis on the SDOH variables were performed. To account for overdispersion, multivariate negative binomial regressions were run while controlling for confounders and testing for significant interactions.

**Results:**

The results of the regression analysis indicated that an increase in Factor 1 (economic stability, education, and health and healthcare determinants) was associated with a statistically significant increase in the number of PLWH with uncontrolled HIV. Additionally, we found a significant interaction between Factor 1 and White race. Among persons of low socioeconomic status, White race is associated with a reduction in PLWH with uncontrolled HIV.

**Conclusions:**

These results suggest that reducing poverty and increasing education and rates of health insurance should result in significant reductions in PLWH with uncontrolled HIV. These results have the potential to influence future policy, interventions for retention, adherence, and continuity of care to improve suppression rates in MDC.

## Introduction

There are over one million people living with HIV (PLWH) in the USA, and in 2017, there were over 38,000 new HIV diagnoses in the country, 52% of which were diagnosed in the South [1]. The Centers for Disease Control and Prevention’s (CDC) 2017 HIV Surveillance Report indicates the Miami, FL, metropolitan statistical area (MSA) had the highest rate of new HIV diagnoses in the country and the highest HIV prevalence rate among adults and adolescents [2]. In the nation, the Miami MSA also had the highest HIV prevalence rate for Black/African American males, the highest rate of new HIV diagnoses among Black/African American females, the second highest rate of HIV diagnoses among Hispanic males, the highest rate of HIV diagnoses among White males, and the highest HIV prevalence rate for White females [2]. Moreover, the Miami MSA had the highest death rate in the nation among PLWH in 2016, as of the latest available data [2].

Miami, FL, has been one of the most impacted cities in the country by the HIV epidemic based on yearly HIV diagnoses over the last 10 years [1, 3–11]. By the end of 2017, there were 28,055 PLWH in MDC; 68.8% was in HIV care, 63.9% was retained in HIV care, and 58.3% was virally suppressed [12]. Consequently, 40.1% of all PLWH in 2017 had uncontrolled HIV, which we define as either being out of HIV care or having a detectable viral load: 31.2% of all PLWH in MDC were out of HIV care and another 8.9% were in care but had a detectable viral load. PLWH with uncontrolled HIV are a potential public health threat because they are highly likely to transmit HIV to their HIV-negative partners, due to the infectiousness that is attributed to having a large viral load [13–15]. Traditionally, patient-level factors have been the core of HIV prevention and treatment interventions; however, there has been a shift toward considering community-level variables such as the social determinants of health (SDOH), which have now been identified as potential factors responsible for health inequities, particularly among PLWH [16, 17]. Such a shift to the SDOH, which encompasses variables associated with economic stability, education, social and community context, health and healthcare, and neighborhood and built environment, may be particularly useful when the number of persons with poor outcomes in a community is high, as is the case in MDC.

According to HealthyPeople 2020, the SDOH are “conditions in the environments in which people are born, live, work…that affect a wide range of health, functioning and quality-of-life outcomes” [18]. It has, therefore, been suggested that improving the conditions of a place where people live can improve the overall health of the people who live there [19]. The SDOH are comprised of five key determinants: economic stability, education, social and community context, health and healthcare, and neighborhood and built environment [18]. There are specific domains within the five determinants that help explain their impact on society. The economic stability determinant is explained by the employment, food insecurity, housing instability, and poverty domains [18]. The education determinant is explained by early childhood education and development, enrollment in higher education, high school (HS) graduation, and language and literacy [18]. The social and community context determinant is explained by civic participation, discrimination, incarceration, and social cohesion [18]. Health and healthcare is explained through access to healthcare, access to primary care, and health literacy [18]. Lastly, the neighborhood and built environment determinant is explained by access to foods that support healthy eating patterns, crime and violence, environmental conditions, and quality of housing [18].

In this study, we focus on the relationship of the SDOH to health outcomes in the HIV community. In an HIV Surveillance Supplemental Report by the CDC analyzing the SDOH among adult PLWH, the rate of HIV diagnoses increased as the percentage of residents living below the poverty level, percentage of residents with less than a HS education, and percentage of residents uninsured increased, and among Hispanics and Latinos, the lowest percentages of viral suppression were found in counties with the lowest poverty, income, education, and health insurance coverage [17]. Due to the alarmingly high number of PLWH with uncontrolled HIV in MDC, and the considerable Hispanic/Latinx demographic, we were interested in exploring possible SDOH conditions that might predict the ZIP codes with the highest number of these PLWH. The purpose of this study was to determine the association between the SDOH and the number of persons with uncontrolled HIV aged 15 years and older in MDC, FL ZIP codes.

## Methods

### Study Design

This is an ecological study, using cross-sectional surveillance data, involving PLWH, aged 15 years and older, who were either out of HIV medical care or had a detectable viral load from January to December 2017 and resided in MDC, FL, ZIP codes. The information on PLWH provided by the Florida Department of Health (FDOH) from their surveillance system was aggregated at the ZIP code level, due to confidentiality laws, and then merged with ZIP code level SDOH data. Of the 80 MDC ZIP codes, only ZIP codes verified for MDC by the US Postal Service and with three or more PLWH were included in the final dataset. Three MDC ZIP codes were excluded, one was a commercial zone with no associated data, and two contained less than three PLWH and thus were considered identifiable. The final sample contained 77 of the 80 MDC ZIP codes, with 27,478 individual PLWH aged 15 years and older living within those ZIP codes. Address level data (e.g., crime data) were geocoded and assigned to the associated ZIP codes (unit of analysis) using ArcGIS version 10.5 to create maps for the variables of interest.

We defined uncontrolled HIV based on the FDOH and HIV/AIDS Bureau Performance Measures provided by the Health Resources and Services Administration (HRSA) as a PLWH who was out of treatment (i.e., did not have viral load test or a CD4 count) or had a viral load greater than or equal to 200 copies/ml [20]. Viral load values were obtained through HIV testing from diagnostic and clinical laboratories that are mandated to report these results to the FDOH and captured in the enhanced HIV/AIDS Reporting System. The exposures were the SDOH, the outcome was the number of PLWH with uncontrolled HIV, and the potential confounders were age, gender, mode of HIV transmission, and race/ethnicity.

### Measures

Data on PLWH were obtained from the FDOH. These data were aggregated to the ZIP code level and included information on following potential confounders: PLWH’s age, self-identified gender, mode of HIV acquisition through categorized types of exposures, and race/ethnicity. Age was grouped into four categories representing five stages of life as used in other related studies: (1)mid/older adolescents and young adulthood (15–24 years), (2) early adulthood (25–44 years), (3) middle adulthood (45–64 years), and (4) late adulthood (65 and over) [21–23]. The FDOH did not provide data on children 15 years of age and younger; thus, this age group was not included in the analysis. PLWH’s gender was classified as either male or female, based on self-identification. In addition, FDOH categorized mode of HIV transmission into five categories: (1) men who have sex with men (MSM), (2) persons who inject drugs (PWID), (3) men who have sex with men and are persons who inject drugs (MSM/PWID), (4) heterosexual contact, and (5) other. The “other” mode of transmission category included HIV infection acquired through perinatal transmission and exposure to infected blood during transfusions. Race and ethnicity were classified as Hispanic, non-Hispanic Black/African American, non-Hispanic White, and other race, which included people of multiple races, Asian, and Pacific Islanders.

To examine the association between the SDOH and the number of PLWH with uncontrolled HIV per MDC ZIP code, we obtained ZIP code level data from the 2013–2017 US American Community Survey (ACS), for people aged 15 years and older, to approximate the SDOH domains based on the five SDOH identified by the HealthyPeople 2020 initiative [18, 24]. At least one domain and a primary variable representing that domain were selected for analysis for each SDOH determinant.

The first determinant, **economic stability**, included the employment domain with the variable employment status and the poverty domain with the variable households below poverty level, government assistance, and median household income.

The second determinant, **education**, was comprised of three domains: HS graduation, enrollment in higher education, and language and literacy. The variables used to represent these domains were categorized into less than an HS education and HS graduation only (HS graduation domain), greater than HS education (enrollment in higher education domain), and language other than English spoken at home (language and literacy domain). Of the “language and literacy” domain, only language was considered relevant to this study due to MDC’s unique demographics in which most of MDC’s population is Spanish speaking.

The third determinant, **social and community context**, used the civic participation and incarceration domains and included the following variables: census return rates by ZIP codes for the civic participation domain, which was provided by the US Census Bureau, and incarceration measured by rate of total incarcerations per population in 2017 within each ZIP code for the incarceration domain, which was provided by the MDC Department of Corrections and Rehabilitation.

The fourth determinant,** health and healthcare**, assessed the access to health or healthcare domain through the variable health insurance rate.

The fifth determinant, **neighborhood and built environment**, was assessed by the domains of violent crime and access to foods. Data on violent crime was obtained from the MDC Central Records Bureau Database via a public records request of the scene address at which the crime/offense occurred. This database contains police reports and records from the Miami-Dade Police Department that occur within MDC. This database includes information on violent crime coded using the Federal Bureau of Investigations uniform crime rates definitions. The uniform crime rates definitions for violent crime included aggravated assault, murder, aggravated stalking, rape, negligent manslaughter, robbery, and police shooting. Access to foods was measured by the percent of households within half-a-mile from a supermarket within each ZIP code, as determined by the US Department of Agriculture Economic Research Service 2015 Food Access Research Atlas [25].

### Analyses

The SDOH variables were converted into proportions, and a principal component analysis (PCA) was conducted to reduce the number of variables. An oblique (promax) rotation was used to account for the high correlation between the variables, and the rotated factor pattern was used to arrive at the principal components. In this study, significant loadings were those with a loading greater than 0.4, and components with an Eigenvalue greater than one, based on the Kaiser value criterion and Scree plots, were retained [26]. Descriptive statistics, such as univariate analyses, which included the distribution and frequency of the data, and bivariate analyses, which measured any potential preliminary associations of the characteristics of PLWH by outcome status, were completed first. To account for overdispersion in this dataset, multivariate negative binomial regressions were then used to model these count data for an association [27, 28]. Testing for significant findings and exposure-confounder interactions to examine any variation of the exposure by level of a confounder was done using an alpha level of 0.05 in SAS 9.4.

Regressions were used to calculate odds ratios and confidence intervals for the association between SDOH and the number of people with uncontrolled HIV while controlling for age, race/ethnicity, gender, and mode of HIV transmission. Age, race/ethnicity, gender, and mode of HIV transmission were included in the analysis a priori because they were the only sociodemographic variables available to the study team. These variables were treated as confounders due to their associations with both the exposure and the outcome. For example, race/ethnicity is associated with most aspects of the SDOH in that minorities, specifically Black/African Americans and Hispanics/Latinx, are substantially poorer and live in less desirable neighborhoods than their non-Hispanic White counterparts. Race/ethnicity is also associated with HIV in that minorities experience disproportionately high incidence of disease and poor health outcomes related to HIV infection, as well as have a higher rate of uncontrolled HIV.

## Results

Of the 27,478 individual PLWH aged 15 years and older included in this analysis, 11,194 (41%) had uncontrolled HIV in 2017. Among all PLWH, 74% are male, 45% are Hispanic, 42% are Black/African American, and 11% are White (see Table 1). Compared with the MDC population, both males and Blacks/African Americans in this study were overrepresented, while Hispanics were underrepresented. The greatest portion of PLWH in MDC were aged 45–64 years (55%), and the most common mode of HIV transmission was MSM (54%). The percentages of PLWH with uncontrolled HIV varied by ZIP code, ranging from 24 to 68%.

**Table 1:** Characteristics of adults and adolescents (15 years and older) living with HIV in Miami-Dade County, FL, 2017

	People living with HIV (*N* = 27,299) *N* (%)	People living with HIV with uncontrolled HIV~(*N* = 11,132) *N* (%)	Range per ZIP code for the number of PLWH with uncontrolled HIV *N*
Gender			
Male	20,361 (75%)	8119 (73%)	7–1865
Female	6938 (25%)	3013 (27%)	0–556
Race/ethnicity			
Hispanic	12,414 (45%)	4202 (38%)	4–1057
Non-Hispanic Black	11,632 (43%)	5572 (50%)	0–1033
Non-Hispanic White	2907 (11%)	1204 (11%)	0–670
Other race	346 (1%)	154 (1%)	0–35
Age group (years)*			
15–24	618 (2%)	275 (2%)	0–43
25–44	8558 (31%)	3553 (32%)	0–632
45–64	15,115 (55%)	5928 (53%)	4–1152
65+	2961 (11%)	1348 (12%)	0–181
HIV transmission category*			
Men who have sex with men (MSM)	14,795 (54%)	5283 (47%)	4–1750
Persons who inject drugs (PWID)	1521 (6%)	817 (7%)	0–144
Men who have sex with men and are persons who inject drugs (MSM/PWID)	670 (2%)	312 (3%)	0–42
Heterosexual contact	9791 (36%)	4422 (40%)	0–711
Other exposure	235 (1%)	156 (1%)	0–32

*Note that when percentiles do not add up to 100, it denotes missing information from among PLWH in this dataset

~ Uncontrolled HIV is defined as either being out of HIV care or having a detectable viral load

The PCA, which included all SDOH variables at the ZIP code level, resulted in three factors. Factor 1 included the education, economic stability, and health and healthcare determinants of the SDOH; Factor 2 included social and community context and neighborhood and built environment determinants; and Factor 3 included education and neighborhood and built environment determinants. Factor 1 values ranged from − 2.29 to 1.94, with the higher positive numbers representing the lowest socioeconomic status (SES), lowest education, and least access to health insurance. Factor 2 values ranged from − 0.72 to 2.62, with the higher positive numbers representing the highest crime rates and lowest civic participation rates. Factor 3 values ranged from − 2.41 to 2.12, with the higher positive numbers indicating the lowest proportion of people with access to foods.

The regressions included the three factors as exposures, the number of PLWH with uncontrolled HIV in each ZIP code as the outcome, the confounders, and a significant interaction between Factor 1 and White race. The unadjusted estimates indicate that Factors 2 and 3 directly affect the number of PLWH with uncontrolled HIV (see Table 2). However, when accounting for confounders, these factors lost significance, and only Factor 1 had a statistically significant effect on the outcome. The regressions suggest that a one unit increase in Factor 1 results in a 16% (1.16 (C.I. 1.07, 1.26)) increase in PLWH with uncontrolled HIV while holding all other variables constant. Similarly, a 1% increase in males produces a 470% (5.70 (C.I. 1.91, 17.02)) increase in PLWH with uncontrolled HIV, and a 1% increase in PLWH aged 25–44 creates a 161% (2.61 (C.I. 1.03, 6.60)) increase in PLWH with uncontrolled HIV. While the unadjusted estimates for the HIV transmission categories were significant, the adjusted estimates showed no significant effects when accounting for confounding factors. The regressions also revealed a significant interaction between Factor 1 and the percentage of Whites in MDC ZIP codes. This interaction term reveals that among those PLWH who are high in Factor 1, being White was associated with a 66% (0.34 (C.I. 0.20, 0.58)) reduction in the number of PLWH with uncontrolled HIV. In other words, people who are White and low SES are significantly less likely to have uncontrolled HIV than the other racial/ethnic groups that are low SES.

**Table 2:** Impact (odds ratios) of sociodemographic characteristics and social determinants of health factors on the number of adults and adolescents (15 years and older) living with uncontrolled HIV in Miami-Dade County, FL, 2017

Parameters	Unadjusted OR estimates	95% CI	AdjustedOR estimates	95% CI
Exposures				
Factor 1	1.04	(0.99, 1.09)	1.16	(1.07, 1.26)*
Factor 2	1.11	(1.03, 1.19)*	0.98	(0.91, 1.04)
Factor 3	0.94	(0.90, 0.99)*	1.05	(0.99, 1.12)
Gender				
Male	0.74	(0.53, 1.04)	5.70	(1.91, 17.02)*
Female	Reference	Reference	Reference	Reference
Race/ethnicity				
Hispanic	0.67	(0.58, 0.78)*	0.35	(0.01, 11.91)
Non-Hispanic Black	1.39	(1.20, 1.61)*	0.46	(0.01, 15.68)
Non-Hispanic White	1.39	(0.79, 2.44)	1.68	(0.04, 70.57)
Other race	Reference	Reference	Reference	Reference
Age group (years)				
15–24	8.52	(0.51, 142.48)	14.35	(0.90, 229.55)
25–44	0.84	(0.39, 1.80)	2.61	(1.03, 6.60)*
45–64	1.44	(0.67, 3.11)	1.42	(0.51, 3.93)
65 and older	Reference	Reference	Reference	Reference
HIV transmission category				
MSM	0.66	(0.52, 0.82)*	0.99	(0.01, 189.56)
PWID	7.15	(3.02, 16.93)*	14.93	(0.09, 2524.25)
MSM/PWID	126.13	(10.16, 1566.34)*	0.23	(0.00, 83.35)
Heterosexual contact	1.42	(1.07, 1.88)*	4.64	(0.03, 785.41)
Other transmission	Reference	Reference	Reference	Reference
Interaction Term				
Factor 1 x NHW			**0.34**	**(0.20, 0.58)**

*OR*, odds ratio

*CI*, confidence interval

*Unadjusted OR estimates,* crude association

*Adjusted OR estimates,* association adjusted for all variables shown, including the interaction term

* Estimate is statistically significant (p-value <0.05)

*Factor 1*. education, economic stability, and health and healthcare determinants

*Factor 2,* social and community context and neighborhood and built environment determinants

*Factor 3,* education and neighborhood and built environment determinants

*Other race*, includes people of multiple races, Asians, and Pacific Islanders

*MSM*, men who have sex with men

*PWID*, persons who inject drugs

*MSM/PWID*, men who have sex with men and are persons who inject drugs

*Other Transmission*, includes perinatal transmission, blood transfusions, and risks not identified

*NHW*, non-Hispanic White

**Fig. 1:**
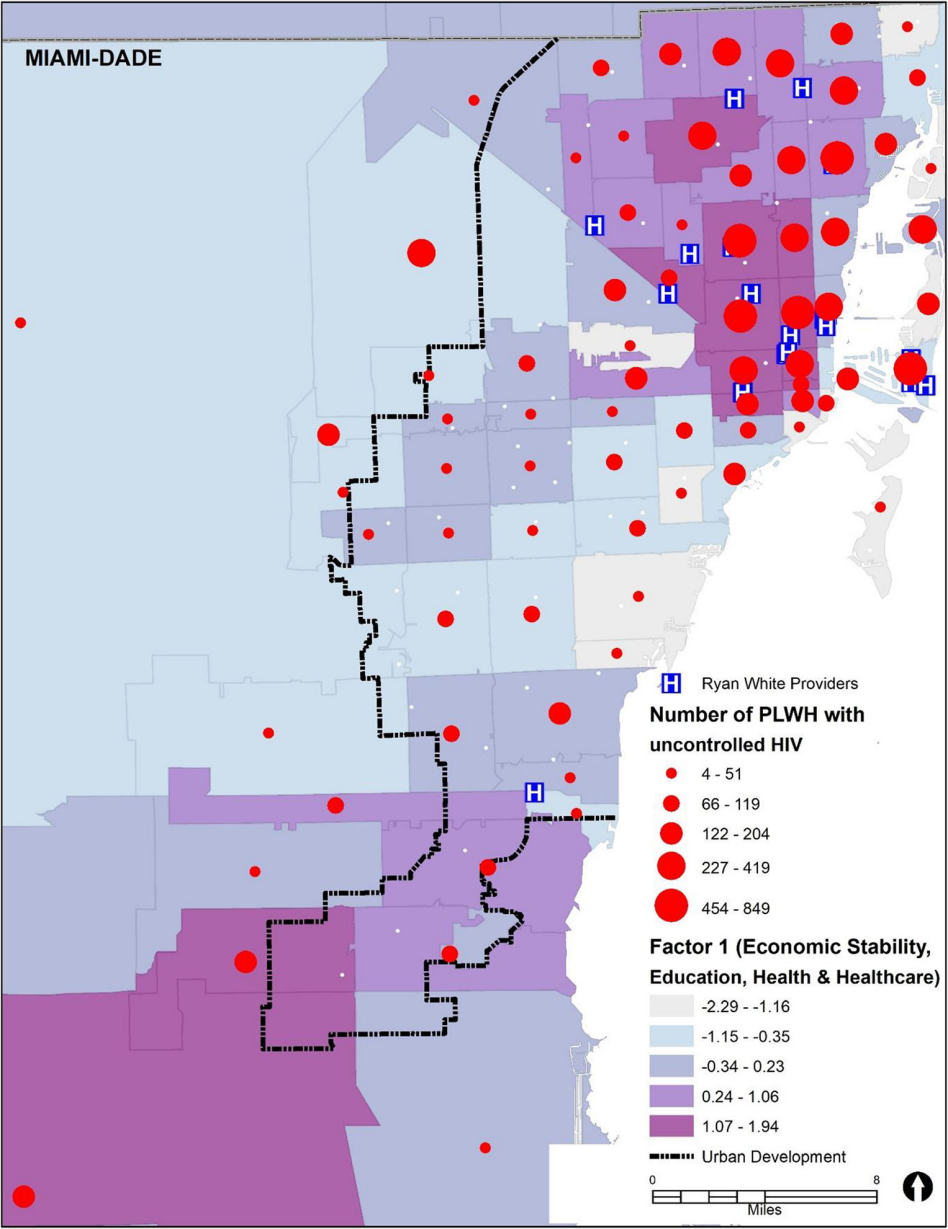
Map of Factor 1 and the number of adults and adolescents (15 years and older) living with HIV with uncontrolled HIV in Miami-Dade County, FL, ZIP codes, 2017. Note that the higher positive numbers of the Factor 1 range (− 2.29 to 1.94) represent the lowest socioeconomic status (SES), lowest education, and least access to health insurance

**Fig. 2:**
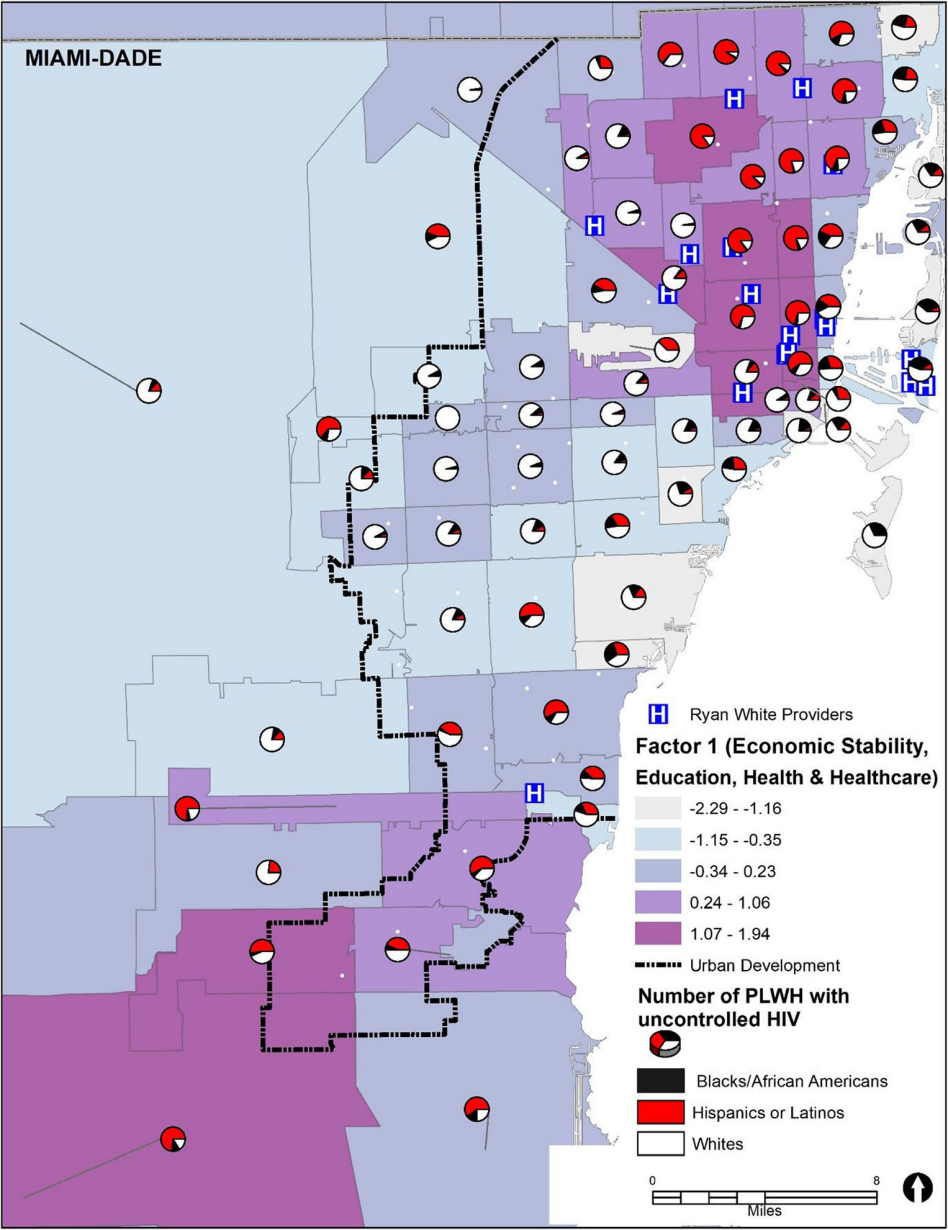
Map of Factor 1 and the proportion of adults and adolescents (15 years and older) living with HIV with uncontrolled HIV among the three racial/ethnic groups in Miami-Dade County, FL, ZIP codes, 2017. Note that the higher positive numbers of the Factor 1 range (− 2.29 to 1.94) represent the lowest socioeconomic status (SES), lowest education, and least access to health insurance

Figure 1 shows the distribution of PLWH with uncontrolled HIV aged 15 years and older regardless of race/ethnicity in each MDC ZIP code by Factor 1 (size of the red dots corresponds to the number of PLWH). As can be seen from Fig. 1, ZIP codes with darker shading denote higher scores in Factor 1, which is consistent with ZIP codes of lower SES. These ZIP codes are also areas with the highest numbers of PLWH with uncontrolled HIV and the highest density of Ryan White providers, which points to some unknown barriers to care that should be further explored. Figure 2 can be compared with Fig. 1, which shows that Black/African American and Hispanic subpopulations reside in ZIP codes with the lowest SES. In contrast, the largest proportions of White PLWH with uncontrolled HIV reside in ZIP codes of higher SES. When comparing the ZIP codes within the urban development line to those outside the urban development line, we can see that ZIP codes outside the urban development line tend be of higher SES. However, more than half of all PLWH in those ZIP codes have uncontrolled HIV, and over 90% of the population in those ZIP codes outside the urban development line are either Black/African American (~ 45%) or Hispanic/Latinx (~ 45%). This suggests that for those people living outside the urban development line, who live in ZIP codes low in Factor 1, there may be other, unknown, factors, along with race/ethnicity, that are contributing to the high rate of uncontrolled HIV in that area.

## Discussion

The purpose of this study was to develop a better understanding of the factors that may be contributing to the extraordinarily high rates of uncontrolled HIV in MDC. Because of the established association between the SDOH and adverse health outcomes, we investigated the potential impact of the SDOH on the high rates of PLWH who are out of care and/or who have a detectable viral load. The findings revealed that 41% of all PLWH living in MDC in 2017 had uncontrolled HIV. Males and Black/African Americans were overrepresented; among these, the most remarkable overrepresentation occurred among Black/African Americans who account for 18.2% of the MDC population, while they represented 42% of the population of PLWH and 54% of the population with uncontrolled HIV. The overrepresentation of Blacks among PLWH is about 170%, whereas the overrepresentation of Blacks among PLWH with uncontrolled HIV is about 205%.

Overall, our findings indicate that of the three factors encompassing the SDOH, Factor 1(economic stability, education, and health and healthcare determinants) was the only factor to reach significance in its effect on increasing the number of PLWH with uncontrolled HIV, after controlling for confounders. Additionally, we found that a 1 % increase in males living with HIV corresponds to a 470% increase in PLWH with uncontrolled HIV. Although these results appear extreme, the population of PLWH in MDC is comprised of mostly males, which is an overrepresentation of the total population in the county, but on par with the overall demographics of the HIV epidemic in the USA [1]. Males make up the bulk of the PLWH, and compared with females, they are less likely to be receiving HIV care [1].

Among the other significant findings, we also found a 161% increase in uncontrolled HIV when there is a 1 % increase among PLWH aged 25–44. Given the age group and the demographics among incident cases, according to the CDC’s Table 1a in the surveillance report, the scale of this increase could be due to these PLWH being new diagnoses and thus having uncontrolled HIV disease at the time of testing [1]. The significant interaction in the model between non-Hispanic Whites and Factor 1 suggests that living in low SES neighborhoods, having low education and/or lack of health insurance, does not have a negative impact on HIV control in non-Hispanic Whites. While being high in Factor 1 is deleterious for the population of PLWH overall in terms of lower rates of HIV control, being a non-Hispanic White is protective for those who are high in Factor 1. These findings are aligned with literature that supports differences in onset and severity of disease between racial minorities and Whites, while under similar social and environmental conditions, most considerably, these differences are seen in terms of HIV morbidity and HIV-related mortality [29, 30].

Overall, this study suggests that for PLWH, poverty, lack of education, and being uninsured have detrimental effects on achieving HIV control [31]. Inversely, reducing poverty and improving education levels and insurance coverage should result in significant reductions in the number of PLWH who are out of HIV medical care or who have a detectable viral load [32]. To achieve favorable results in HIV control, new interventions that address barriers to improving healthcare access and insurance coverage, as per the health and healthcare determinant of the SDOH, should be implemented. Chronic disease research has shown that interventions utilizing community health workers in underserved populations, particularly racial/ethnic minorities, have had positive effects on health outcomes in terms of prevention and reduced disease progression [33–37]. For both Black/African Americans and Hispanic/Latinx, the use of patient navigators has been shown to be a successful intervention that has had significant effects on improving timely linkage to care, retention in care, and preventing declines in viral suppression [38–41].

Nevertheless, the results of this study have the potential to influence current and future policy regarding the need for additional interventions for retention, adherence, and continuity of care to improve treatment adherence and viral load suppression in MDC. If in fact, communities high in Factor 1 (lowest SES, lowest education, and least access to health insurance) are most likely to have high rates of uncontrolled HIV, then we would propose that effective HIV healthcare delivery may need to involve community-based participatory approaches to become more comprehensive with wrap around services from patient navigators to culturally diverse community health workers to medical-legal partnerships, for those of low SES, low education, and low access to health insurance, specifically for Black/African Americans and Hispanic/Latinx in MDC [33–36, 38, 39, 41–50].

An example of a program that could benefit from the results of this study is the MDC “Getting to Zero” HIV/AIDS Initiative, which was created in response to the US Department of Health and Human Services’ Ending the HIV Epidemic: A Plan for America. The “Getting to Zero” Initiative created a set of recommendations for the county that would eventually lead to a reduction in HIV incidence through a focus on prevention and treatment. This research could benefit their recommendations by providing guidance on the areas where certain campaigns should be concentrated. For example, their first recommendation was to provide comprehensive sex education throughout the MDC public school system. Though this recommendation could prove very effective, the approval process for this task will be lengthy, and the dissemination of materials and systems changes throughout the school system may be just as time-consuming. Using the results of this study, we would propose that once this plan is approved, they focus their efforts on setting up these modified sex education programs in schools that are in ZIP codes high in Factor 1, as per our findings, because these students are more likely to become the most at-risk adults.

There are several inherent limitations in this study that should be addressed. The cross-sectional nature of this research does not allow for causal inferences to be made, and the ecological design could present issues with interpretation of the results. To avoid the ecological fallacy, it is important to note that the results of this study reflect ZIP codes within MDC, not individual PLWH. Additionally, although ZIP codes have been found to be an adequate, though not perfect, proxy for individual SES and have been used with good effect in similar studies, as a geographic unit of analysis, ZIP codes lack standardization and are dynamic in structure [51–53]. The data also only included PLWH 15 years of age and older, and thus, this study is only generalizable to that age population. The data did not contain country of origin information, which is of great interest in this community, because foreign-born make up a large proportion of the population of Miami-Dade County [24]. Specifically, it is of great interest in the Venezuelan immigrant population, which has been steadily increasing in MDC, and who may have been affected by sporadic drug shortages in their home country. Additionally, the data did not identify incident cases of PLWH. These new cases would inherently fall into the detectable or out of care category if they were diagnosed toward the end of the year, not giving them enough time to effectively link to care. Moreover, although the use of multiple publicly available datasets is a major strength of this study, it also has certain innate limitations in terms of how the data was collected, as different methods were used, the time frames for when the data were collected, as this also varied between datasets, and the populations from which data were obtained, as some datasets used people over 15 years of age and others used people over 18 years of age.

We used HIV surveillance, ACS, violent crime, incarceration, census return, and USDA data to evaluate the association between the SDOH and the number of PLWH with uncontrolled HIV in MDC ZIP codes. In the South, there are a great number of PLWH with uncontrolled HIV, which is one of several factors that may contribute to the high rate of new HIV diagnoses every year. Further research is needed to determine if the high rate of incident cases in other Southern communities may also be resulting from the impact of the SDOH and to establish why areas saturated with HIV care providers continue to experience poorer health outcomes. If so, the most effective way of reducing new diagnoses would be to improve rates of engagement into treatment, retention in treatment, and adherence in a way that incorporates specific strategies to address the SDOH.
